# Combined Toxicological Effects of Di (2-Ethylhexyl) Phthalate and UV-B Irradiation through Endoplasmic Reticulum Stress—Tight Junction Disruption in Human HaCaT Keratinocytes

**DOI:** 10.3390/ijms23147860

**Published:** 2022-07-16

**Authors:** Yong Sun Lee, Hyo-Jeong Hwang, Yean-Jung Choi

**Affiliations:** 1 College of Pharmacy and Medical Research Center, Chungbuk National University, Cheongju 28160, Korea; kallintz@gmail.com; 2 Department of Food and Nutrition, Sahmyook University, Seoul 01795, Korea; hjhwang@syu.ac.kr

**Keywords:** Di (2-ethylhexyl) phthalate, UV-B irradiation, ER stress, TJ disruption, keratinocytes

## Abstract

Di (2-ethylhexyl) phthalate (DEHP) is widely used as a plasticizer, and human exposure to DEHP is widespread and frequent. However, information about the combined effect of DEHP and ultraviolet (UV)-B on the skin are still limited. We investigated the cytotoxic effects of DEHP and UV-B on HaCaT keratinocytes and evaluated the related underlying mechanisms involving endoplasmic reticulum (ER) stress signals and the disruption of junction complexes as an effective target for skin inflammation. Our results revealed that co-treatment with DEHP and UV-B irradiation alleviated the cell cytotoxicity and markedly decreased X-box binding protein 1 (XBP1), endoplasmic reticulum oxidoreductase 1 alpha (Ero1α), and C/EBP homologous protein (CHOP) whereas a single dose of 40 mJ/cm^2^ UV-B generated mild ER stress to slightly less or similar levels as that seen with DEHP. DEHP was also shown to inhibit tight junctions (TJs) after UV-B irradiation, increased apoptosis by altering apoptotic gene Bax and stress kinases, JNK, and p38 MAPK. Furthermore, exposure of HaCaT cells to DEHP and UV-B irradiation resulted in the marked suppression of the nuclear factor kappa B (NF-κB)/p65 signaling pathway. Taken together, our data suggest that nontoxic DEHP and UV-B irradiation regulated ER stress and epidermal TJ disruption with the induction of apoptosis activation and the secretion of proinflammatory cytokines such as interleukin 1 beta (IL-1β) and IL-6 in human keratinocytes. Further investigation is needed to confirm the mechanisms implicated in its toxicity and determine the effects of exposure to DEHP and UV-B irradiation on markers involved in this study.

## 1. Introduction

Health hazards posed by phthalates have been brought to the public’s attention due to their potential toxic effects. The annual global production of phthalates is estimated at around 18 billion pounds and above 2 million tons of DEHP alone, the most abundant phthalate, as they are widely used in plastic products to improve the flexibility and durability [1]. Although the toxicity of phthalates remains under debate, a large variety of studies have revealed high exposure of DEHP in urine and blood, as well as semen, cord blood, and breast milk [2,3,4,5,6], and the apparent toxicity of DEHP affecting human health [7,8]. Several studies indicate that the daily intake of phthalates or phthalic acid esters may reach values as high as 70 μg/kg/day, based on various mechanisms including dermal exposure [9,10,11,12,13].

The skin functions as an immune organ against outside chemicals and pathogens [14,15]. Several reports have indicated that UV irradiation can activate components of the cutaneous immune system and precipitate immune-mediated diseases by a variety of mechanisms [16,17,18]. The midrange ultraviolet radiation (UV-B) is the most relevant with respect to physical injury to human skin and causes severe damage including cutaneous inflammation. Studies on UV exposure have been conducted worldwide, but few studies have provided definitive UV doses [19,20,21]. In a study that directly measured individual UV exposure, students were exposed to 1 MED (minimum erythema dosage) and 2 MED of solar UV per day, respectively [22]. They considered the maximum daily UVB dose to be 7 KJ/m^2^ and used 22 MED as the maximum daily UVB dose. In addition, since 1 MED was distributed at 20–60 mJ/cm^2^, 200 MED was determined as the annual average dose. Importantly, 99% of UV-B radiation is absorbed within the outermost 0.03 mm of the epidermis when searching for physiological targets. Accordingly, UV-B-induced keratinocytes probably constitute a major cellular target [23,24]. The major risk factor for skin disorders is exposure to UV radiation from sunlight, but other environmental exposures may also play a role in combination with UV. Previous studies showed that the combined exposure of human keratinocytes to an environmental pollutant benzo[a] pyrene and UV caused a more than additive increase in DNA damage compared with each agent alone [25,26,27]. However, the synergistic or additive phototoxic effect of phthalates on the skin remains unclear.

Alterations in endoplasmic reticulum (ER) homeostasis can cause a cellular stress response known as the unfolded protein response (UPR) in response to the accumulation of misfolded or unfolded proteins. When the ER stress is extensive or sustained and the function of ER cannot be restored, the UPR can eventually prompt cell death. Many studies have indicated the role of ER stress and the activation of the UPR in the development of pathologic conditions such as apoptosis and inflammation [28,29,30]. One study showed a causal relationship between ER stress and DEHP exposure [31], but no sufficient data are available in this respect concerning both DEHP and UV-B irradiation.

The epidermal tight junction (TJ) barrier is an important structure to maintain the integrity of the skin barrier with the stratum corneum (SC) [32]. Recent studies have discussed the interaction of TJs with several skin diseases or skin barrier alterations [33,34]. A more recent study suggested that the epidermal barrier becomes more permeable in response to UV, and the mechanisms may include a reduction in the TJ function in the skin [35]. Another study reported that UV-B and oxidative stress could induce the elevation of permeability mediated by claudin-1 in HaCaT cells [36]. Moreover, both barrier perturbation and UV-B irradiation induce ER stress and UPR in keratinocytes [37,38]. Similarly, the most recent study showed that ER calcium depletion disrupted TJ barrier integrity, and the IRE1α-XBP1 pathway of the UPR is essential to enhance or maintain the TJ barrier in response to ER stress induced by UV-B irradiation in keratinocytes [39]. However, until now, no study had directly investigated the differential regulation of ER stress and the epidermal TJ barrier in the skin by the combined exposure to DEHP and UV-B irradiation.

The aim of the present study was to clarify new mechanistic insights into the cytotoxic activity of the combined exposure to both DEHP and UV-B irradiation in HaCaT keratinocytes. We tested whether (1) DEHP could induce a synergistic effect on ER stress and TJ disruption with UV-B irradiation; (2) this synergic effect could be sufficient to alter the activation of apoptosis and stress kinases; and (3) the combination treatment could suppress the nuclear factor kappa B (NF-κB)/p65 and NF-E2-related factor 2 (Nrf2) antioxidant signaling with the secretion of proinflammatory cytokines.

## 2. Results

### 2.1. Effect of DEHP, UVB, and Co-Treatment with DEHP and UV-B on Cell Viability of Human Keratinocytes

**Figure 1:**
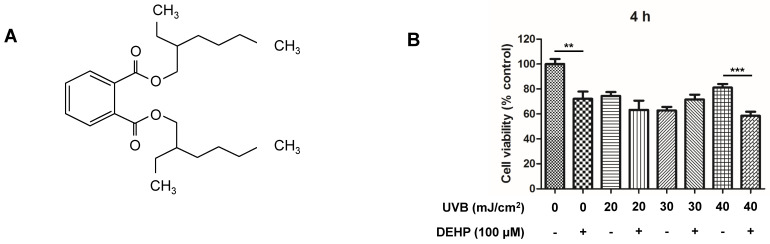
Chemical structure of DEHP (**A**), cell viability in DEHP-treated HaCaT cells challenged with UV-B irradiation at the indicated intensities (**B**). Confluent HaCaT cells were pre-treated with 100 μM DEHP prior to incubation for 4 h with 40 mJ/cm^2^ UV-B. Cell viability was measured using MTT assay and presented as means ± SEM from three independent experiments with multiple estimations. ** *p* < 0.01; *** *p* < 0.001 vs. control group.

To examine the cytotoxic effects of DEHP, UVB, and the co-treatment with DEHP and UV-B, we performed an MTT assay. After 4 h of 100 μM DEHP treatment, HaCaT cells produced mild toxicity, and the co-treatment with 20 to 40 mJ/cm^2^ UV-B alleviated the cell cytotoxicity up to 60% (Figure 1B). Based on these data, the highest intensity of DEHP (100 μM) and UV-B (40 mJ/cm^2^) was selected for subsequent studies, respectively.

### 2.2. Combined Effects of DEHP and UV-B in Acute ER Stress Responses and Tight Junction Disruption

**Figure 2:**
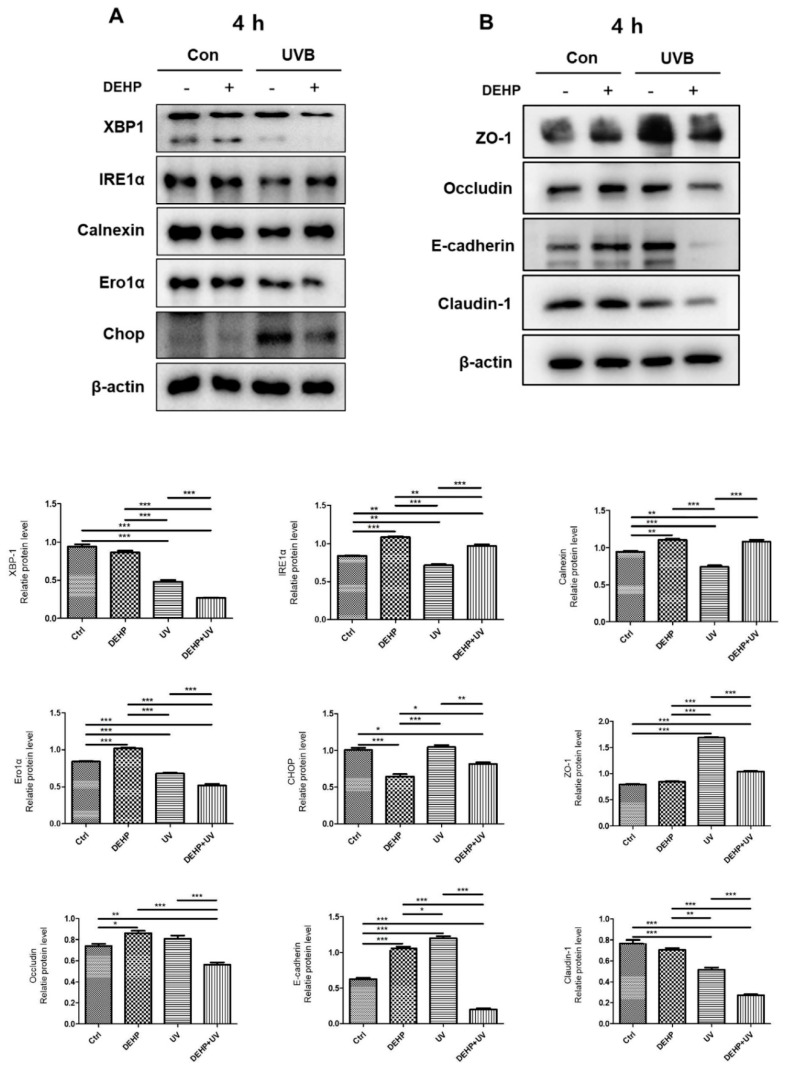
Effect of DEHP on expression of acute ER stress responses and tight junction disruption in UV-B-irradiated HaCaT cells. Cells were treated with 100 μM DEHP and stimulated with 40 mJ/cm^2^ UV-B. Cell lysates were prepared for Western blot analysis with a primary antibody against XBP-1, IRE1α, calnexin, Ero1α, and CHOP (**A**) and ZO-1, Occludin, E-cadherin, and Claudin-1 (**B**). Representative blot data were obtained from three experiments, and β-actin protein was used as an internal control. The bar graphs (means ± SEM) in the bottom panels represent quantitative results of upper blots. * *p* < 0.05; ** *p* < 0.01; *** *p* < 0.001 vs. control group.

We next investigated the potential involvement of ER stress signals and tight junction alterations in the DEHP and UV-B co-treated HaCaT cells. As shown in Figure 2A, the levels of the major ER stress markers XBP1, IRE1α, calnexin, Ero1α, and CHOP were assessed by Western blot analysis. UV-B (40 mJ/cm^2^) induced slight decreases of XBP1, IRE1α, calnexin, and Ero1α compared with control groups. Co-treatment with 100 μM DEHP and UV-B markedly decreased XBP1, Ero1α, and CHOP, whereas IRE1α and Calnexin levels were slightly up, as seen with DEHP. Then, we investigated the potential involvement of ER stress responses in the UV-B-induced TJ alteration in HaCaT. Co-treatment with DEHP and UV-B led to significant decreases in the expression of ZO-1, Occludin, E-cadherin, and Claudin-1 in HaCaT cells, whereas the TJs examined in this study were not significantly disrupted by DEHP alone (Figure 2B).

### 2.3. Combined Effect of DEHP and UV-B on Induction of Apoptosis-Related Proteins and MAPK Signaling

**Figure 3:**
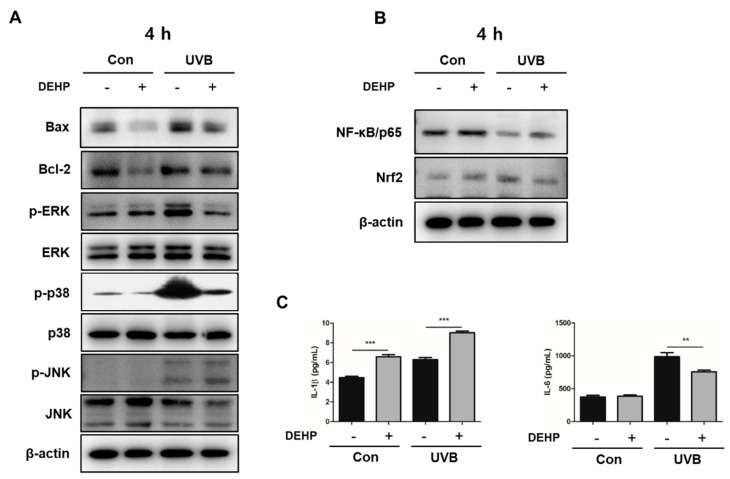

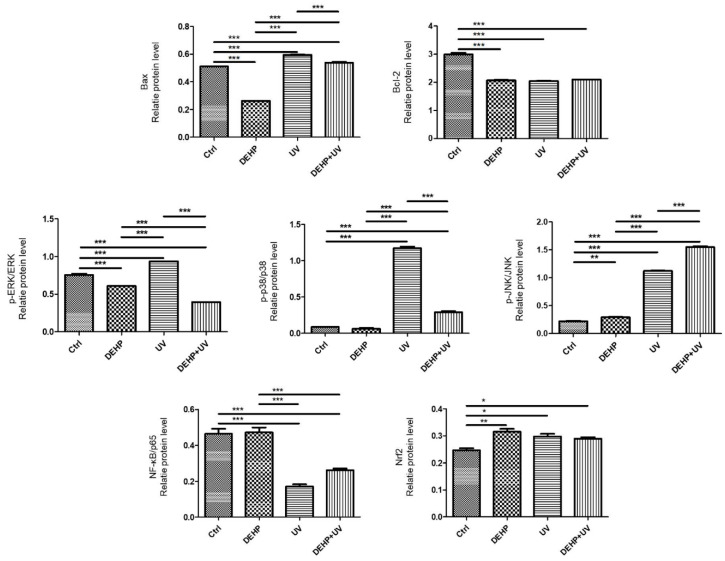
Effect of DEHP on apoptosis-related proteins, phosphorylation of ERK, p38 and JNK, suppression of NF-κB/p65 and Nrf2, and signaling and induction of proinflammatory cytokines in 40 mJ/cm^2^ UV-B-irradiated HaCaT cells. Western blot analysis was carried out with a primary antibody against Bax, Bcl-2, p-ERK, ERK, p-p38, p38, p-JNK, JNK, or β-actin (**A**) and NF-κB/p65 and Nrf2 (**B**). Bands are representative of three independent experiments. The bar graphs (means ± SEM) in the bottom panels represent quantitative results of upper blots. The production of IL-1β and IL-6 (**C**) was measured by ELISA (n = 6 for each group). The results are expressed as the means ± SEM. * *p* < 0.05; ** *p* < 0.01; *** *p* < 0.001 vs. control group.

To determine whether the MAPK signaling pathway was activated by the co-treatment of DEHP and UV-B, the levels of p-ERK, p-p38, and p-JNK were detected by Western blot analysis. As shown in Figure 3A, UV-B (40 mJ/cm^2^) induced significant increases of pro-apoptotic Bax, p-ERK, p-p38, and p-JNK, whereas the pathways examined in this study were not activated by DEHP itself. The co-treatment of DEHP and UV-B partially maintained the induction of the expression of Bax and p-JNK. 

### 2.4. Combined Effect of DEHP and UV-B on NF-κB/p65 Activation, Nrf2 Antioxidant Signaling, and Proinflammatory Cytokine Production

To investigate changes in signal transduction pathways possibly induced by DEHP, UV-B, and co-treatment with DEHP and UV-B, we performed Western blot analysis and ELISA assay. As shown in Figure 3B, UV-B alone and co-treatment with DEHP and UV-B significantly induced the suppression of NF-κB/p65 activation compared to the control group that was not irradiated with UV-B and DEHP itself, respectively. However, DEHP itself did not show a significant difference from the control group. In addition, the Nrf2 expression was obviously induced by the exposure to both DEHP and UV-B. Subsequently, DEHP alone and co-treatment with DEHP and UV-B also induced the activation of proinflammatory cytokines IL-1β and IL-6 compared to the control group after 24 h (Figure 3C).

## 3. Discussion

**Figure 4:**
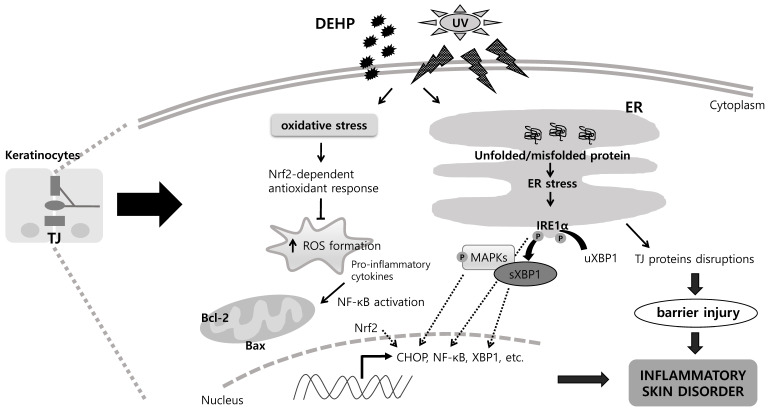
Schematic diagram showing the combined effects of DEHP and UV-B irradiation in HaCaT keratinocytes. As depicted, DEHP and UV-B induced ER stress results in IRE1-mediated development of pathologic conditions such as apoptosis and inflammation, which in turn contributed to TJ disruption. The → indicates activation or induction, and ⊣ indicates inhibition or blockade.

Seven major findings were extracted from this study. (1) Nontoxic 100 μM DEHP induced a synergistic cytotoxic effect with UV-B irradiation at the highest intensity of 40 mJ/cm^2^ after 4 h. (2) DEHP treatment induced the regulation of the ER stress responses (XBP1, Ero1α, and CHOP) with UV-B irradiation. (3) DEHP disrupted TJ proteins (ZO-1, Occludin, E-cadherin, and Claudin-1) with UV-B irradiation. (4) Co-treatment with DEHP and UV-B altered pro-apoptotic Bax and anti-apoptotic Bcl-2 expression. (5) Co-treatment with DEHP and UV-B induced the MAPKs signaling pathways in p-p38 and p-JNK but not in p-ERK. (6) Co-treatment with DEHP and UV-B suppressed NF-κB/p65 activation but increased the Nrf2 antioxidant response. (7) Co-treatment with DEHP and UV-B increased proinflammatory cytokine, IL-1β and IL-6, production after 24 h. Therefore, DEHP and UV-B irradiation resulted in the ER stress-mediated development of pathologic conditions through pathways involving the activation of the ER IRE1α and Nrf2 by interfering NF-κB/p65, which in turn contributed to TJ disruption in keratinocytes (Figure 4). It is difficult to evaluate the effects of phthalates on public health, because several parameters should be taken into consideration. Previous studies tried to establish a systemic approach to the cutaneous toxicity of phthalates [40,41]. We used human keratinocytes to evaluate the phototoxicity of DEHP to the skin, and the experimental results presented in this study possess clinical relevance for finding effective biomarkers for DEHP and UV-B toxicity. Further investigation is needed to confirm the mechanisms implicated in its toxicity and determine the effects of exposure to DEHP and UV-B irradiation on markers involved in this study.

Here, we found that a single dose of 40 mJ/cm^2^ UV-B generated mild ER stress to slightly lower levels than or similar levels to those seen with DEHP. However, co-treatment with DEHP and UV-B markedly decreased the expression of XBP1 and CHOP in this study. Furthermore, our results showed that the co-treatment of DEHP and UV-B disrupted specific TJ proteins including ZO-1, Occludin, E-cadherin, and Claudin-1 whereas the treatment of HaCaT with DEHP or UV-B itself at the concentration used in these experiments did not affect the TJ molecules. The present study did not determine the inhibitory effect on IRE1α phosphorylation in the presence of DEHP and/or UV-B irradiation; however, a recent study indicated that the UV-B-induced activation of the IRE1α-XBP1 arm of UPR can maintain TJ integrity and IRE1α-XBP1 activation plays a protective role in the maintenance of TJ integrity in response to mild ER stress [39].

We then investigated the potential involvement of the direct apoptotic signaling, including Bax and Bcl-2, in this study. Our results show that UV-B irradiation at 40 mJ/cm^2^ induced pro-apoptotic Bax and MAPK signaling pathway in HaCaT at 4 h post irradiation. In addition to the co-treatment of DEHP and UV-B apparently decreased the induction of the expression of Bcl-2. There is emerging evidence revealing that the activated UPR facilitates cell survival in an attempt to relieve ER stress and restore homeostasis; however, prolonged ER stress also induces apoptosis [42,43]. One investigation shows that Bcl-2 knockout increased UV-B-induced apoptosis suggesting the antioxidant activity of Bcl-2 [44]. Moreover, studies have demonstrated that CHOP induces the expression of pro-apoptotic genes, such as DR5, TRB3, BIM, and PUMA, and represses the expression of bcl-2, which triggers apoptosis during ER stress.

In this study, DEHP alone did not induce significant p-p38, but combined exposure significantly induced p-p38 and p-JNK compared with the control cells. It has been reported that the stress kinases JNKs and p38 are functionally linked to the regulation of pro-apoptotic and pro-inflammatory pathways in response to UV-B irradiation. A dominant role of p38 MAPK in the signal leading to Bax activation is also supported in the spontaneously transformed keratinocytes. Consequently, the exact mechanism underlying p38 MAPK-mediated Bax activation may be indirectly involving the regulation of the anti-apoptotic activity of Bcl-2 members through a p38 MAPK-directed phosphorylation. On the other hand, studies of human skin have suggested that low-dose UV-B irradiation induces the rapid activation of stress kinases, JNK and p38 MAPK, which may act to prevent the onset of skin photocarcinogenesis, as they contribute to the elimination of potentially cancerous cells [45].

Another key effector of UV-B generated ROS is the transcription factor NF-κB, which is a well-documented downstream target of the MAPK signal transduction pathway and is, together with the p38 MAPK pathway, a major regulator of inflammation in irradiated skin. Recent studies suggested that UV-B irradiation activates NF-κB, which functions as a survival signal and is distinct from the transcriptional profile induced by cytokine stimulation [46]. Moreover, previous in vitro studies reported that UV-B doses not exceeding 10 mJ/cm^2^ cause several cytoprotective mechanisms, including the activation of the transcription factor NF-E2-related factor 2 (Nrf2) and the induction of anti-apoptotic factors in keratinocytes [47,48]. Consistent with previous studies, co-treatment with DEHP and UV-B suppressed NF-κB/p65 activation and increased Nrf2 antioxidant response. This study also showed that DEHP and UV-B-induced UPR and NF-κB pathways are involved, which in turn induces the secretion of a variety of inflammatory mediators including IL-1β and IL-6. A current study showed that UV-B induces inflammasome activation and IL-1β secretion, providing insight towards potential targets against UV-B-induced inflammation [49]. Further studies are needed to confirm that IRE1α-mediated NF-κB and JNK activation, which leads to the increased expression of pro-inflammatory genes.

## 4. Materials and Methods

### 4.1. Cell Culture

HaCaT cells, spontaneously immortalized human keratinocytes, were purchased from Cell Line Service (DKFZ, Eppelheim, Germany) and were grown in a Roswell Park Memorial Institute 1640 medium (RPMI 1640) supplemented with 10% fetal bovine serum (FBS), penicillin (100 units/mL), and streptomycin (100 μg/mL) at 37 °C in a humidified atmosphere of 5% CO_2_ air. RPMI 1640, penicillin, streptomycin, and FBS were purchased from Gibco Life Technologies (Grand Island, NY, USA). Cells were regularly screened for mycoplasma contamination using Plasmo Test^TM^ (InvivoGen, San Diego, CA, USA).

### 4.2. Treatment with DEHP

DEHP (CAS number: 117-81-7) was purchased from Tokyo Chemical Industry (Tokyo, Japan) and dissolved in DMSO. Cells were seeded 24 h prior treatment. For the experiments, DEHP was treated to give the final concentration of 100 μM for 2 h. The doses of DEHP were selected according to our preliminary dose-finding study, which conducted a cytotoxicity assay and indicated that treatment of DEHP at the concentrations (1–100 μM) and times tested did not largely affect the viability of control or UV-B-irradiated HaCaT cells (data not shown). Equal amounts of DMSO were added to the control groups.

### 4.3. Exposure of HaCaT Cells to UV-B Radiation

The HaCaT cells were exposed to UV-B radiation using a Vilber Lourmat (VL-215 with 2 × 15 W UVB bulbs). The UV-B radiation intensity was measured with a UV light meter (UV-340; Lutron, Taiwan, Taipei). The cells were washed in phosphate-buffered saline (PBS) prior to exposure to UV-B irradiation (20–40 mJ/cm^2^) without lids on the plates. Non-exposed control samples were maintained in the dark under the same conditions. Following exposure to UV-B irradiation, fresh media was added to the cells, and the cells were incubated for 4 h.

### 4.4. Cell Viability

The cell-viability assay was performed as described previously [50]. HaCaT cells were incubated with the indicated concentrations of DEHP for 2 h. The DEHP pre-treated HaCaT cells were then exposed to 20–40 mJ/cm^2^ of UV-B irradiation followed by incubation for 4 h. Cell viability was determined using 3-(4,5-dimethylthiazol-2-yl)-2,5-diphenyltetrazolium bromide (MTT) solution (5 mg/mL).

### 4.5. Western Blotting

The Western blot analysis was performed as described previously [50]. The membranes were incubated with the following primary antibodies: XBP1, IRE1α, Calnexin, Ero1α, CHOP, ZO-1, Occludin, E-cadherin, Claudin-1, p-ERK, ERK, p-JNK, JNK, p-p38, and p38 (1:1000 dilutions; Cell Signaling, Beverly, MA, USA); NF-κB/p65 (1:1000 dilutions; Abcam, Cambridge, MA, USA); Nrf2, Bax, Bcl-2, and β-actin (1:1000 dilutions; Santa Cruz Biotechnology, CA, USA). The blots were analyzed using West-Zol Plus and a molecular imager Fusion FX VilberLourmat (Vilber, Marne-la-Vallée, France).

### 4.6. ELISA

The levels of human recombinant IL-1β, IL-6, IL-8, and TNF-α released from the DEHP-treated cell cultures were measured 4 or 24 h post irradiation. Immediately after UV exposure, fresh media was added to the cells. The levels of cytokines in these media samples were determined using an Ezway Cytokine ELISA Kit (KOMA Biotechnology, Seoul, Republic of Korea).

### 4.7. Statistical Analysis

The results obtained in this study were expressed as the mean ± SEM from at least three independent experiments. The statistical significance was assessed using an unpaired one-way analysis of variance (ANOVA) followed by a Bonferroni post hoc test. The statistical analysis was performed using GraphPad Prism 5.0 software (GraphPad Software Inc., San Diego, CA, USA). *p* < 0.05 was considered statistically significant.

## 5. Conclusions

These findings suggest that there are multiple potential targets of DEHP- and UV-B-induced toxicity through the differential regulation of ER stress and epidermal TJ barrier by altering pro-apoptotic Bax and stress kinases, JNK, and p38 MAPK with NF-κB/p65 activation and the Nrf2 antioxidant response in HaCaT keratinocytes. Further studies are needed to clarify these possibilities.

## Data Availability

The data used to support of this study are included with the article.
